# Agronomic performance of insect-protected and herbicide-tolerant MON 89034 × TC1507 × NK603 × DAS-40278–9 corn is equivalent to that of conventional corn

**DOI:** 10.1080/21645698.2017.1301331

**Published:** 2017-03-31

**Authors:** Denise T. Rezende de Cerqueira, Ariane C. Schafer, Brandon J. Fast, Rod A. Herman

**Affiliations:** a Regulatory Sciences - Dow AgroSciencies Sementes e Biotecnologia Brasil LTDA, Rodovia, Cravinhos, SP, Brazil; b Regulatory Sciences -Dow AgroSciences LLC, Indianapolis, IN, USA

**Keywords:** agronomic characteristics, agronomic equivalence, corn (*Zea mays*), transgenes, transgenic crops

## Abstract

Agronomic characteristics of genetically modified (GM) MON 89034 × TC1507 × NK603 × DAS-40278–9 (PowerCore™ Enlist™), MON 89034 × TC1507 × NK603 (PowerCore™), and DAS-40278–9 (Enlist™) corn, a non-GM near-isogenic hybrid, and 2 commercial non-GM hybrids were assessed in a field study to determine if the agronomic performance of the GM corn hybrids is equivalent to that of non-transgenic hybrid corn. The MON 89034 × TC1507 × NK603 × DAS-40278–9 hybrid corn was developed through stacking of 4 individual transgenic events, MON 89034, TC1507, NK603, and DAS-40278–9 by traditional breeding and contains the *cry1A.105* and *cry2Ab2* (MON 89034), *cry1F* and *pat* (TC1507), *cp4 epsps* (NK603) and *aad-1* (DAS-40278–9) transgenes. These transgenes encode the proteins Cry1A.105, Cry2Ab2, and Cry1F, which confer insect resistance, PAT, CP4 EPSPS, and AAD-1, which confer herbicide tolerance. The following agronomic characteristics were assessed in the study: initial and final stand count, seedling vigor, time to silk, time to pollen shed, pollen viability, plant height, ear height, stalk lodging, root lodging, days to maturity, stay green, disease incidence, insect damage, herbicide injury, and yield. The agronomic assessment was conducted in 2 regions of Brazil (Indianopolis-MG; Cravinhos-SP). The agronomic attributes for all GM entries were statistically indistinguishable from the non-GM near-isogenic hybrid. In addition, most of the agronomic assessments fell within the range of the commercial varieties included in the study. Taken together, MON 89034 × TC1507 × NK603 × DAS-40278, MON 89034 × TC1507 × NK603, and DAS-40278–9 were found to be agronomically equivalent to non-GM corn.

## INTRODUCTION

Producing a sufficient quantity of food will become increasingly challenging as the world's population grows to an estimated 9 billion people by 2050. To supply the estimated 70% increase in the world's food needs that will occur as a result of this population growth, it is imperative that the use of current agricultural technologies is maximized and that new technologies are also developed (FAO, 2014).

Globally, farmers are seeking ways to increase crop production while reducing the use of resources. Genetically modified (GM) crops are a vital tool for increasing productivity while also reducing the impact of agriculture on the environment. The productivity of crops grown for human consumption is negatively impacted by weed, pathogen, and arthropod/animal pests. While crop losses due to these harmful organisms may be substantial, they can be prevented or reduced through crop protection measures. For corn, estimates on potential and actual losses due to pests, even with current crop protection practices, are given to be around 31% (Oerke, 2006).

According to Brookes and Barfoll (2014) and, James (2013) some of the key contributions of GM crops to sustainability include increased crop productivity, improved conservation of biodiversity, reduction in agriculture's ecological footprint, and mitigation of climate change, all of which consequently contribute to the alleviation of poverty and hunger. In 2013, GM crops were grown in 27 countries on an estimated 175.2 million hectares, and more than 90 percent of the 18 million farmers growing GM crops today are resource poor and farm on small plots of land. Using GM crops allows these farmers to increase yields while also decreasing production costs (James, 2013). The vast majority of land where GM crops are produced is planted to corn, soybeans, cotton, and canola with Bt-based insect protection and/or herbicide tolerance traits (Kershen and Fedoroff, 2014). For any new crop variety (GM or non-GM) to be adopted by producers, its agronomic performance must meet or exceed that of the varieties that are currently being used.

Reported here are agronomic characterization results required by some regulatory agencies (in addition to crop composition data) to assess the potential for unintended effects. These agronomic data are collected in addition to the agronomic characterizations that are routinely done by plant breeders for any new variety or hybrid to assess commercial potential.

## MATERIALS AND METHODS

Agronomic assessments were conducted in Indianopolis-MG and Cravinhos-SP Brazil on initial and final stand, seedling vigor, time to silk, time to pollen shed, pollen viability, plant height, ear height, stalk lodging, root lodging, days to maturity, stay green, disease incidence, insect damage, herbicide injury, and seed yield (Table 1). The purpose of monitoring insect damage and disease incidence is only to ensure that these factors had no undue influence on the study results; these data were not collected to evaluate the efficacy of the product. The disease species that were observed in the disease incidence assessments included leaf spot (*Cercospora* spp.), common rust (*Puccinia sorghi*), Turcicum Leaf Blight (*Exserohilum turcicum*), stain diplodia (*Diplodia maydis*), and white spot (*Phaeosphaeria maydis*).

**TABLE 1.** Agronomic characteristics, scale and evaluation timing.

Agronomic characteristics	Scale	Evaluation timing
Initial stand count	Number of plants in 2 rows of each plot	V4
Final stand count	Number of plants in 2 rows of each plot	R6
Seedling vigor	Scoring range 1 3 (1 = poor vigor, 3 = excellent vigor)	V4
Time to pollen shed	Number of days from planting to the day when 50% of plants were shedding pollen	R1
Time to silk	Number of days from planting to the day when 50% of plants had emerged silks	R1
Disease incidence	1–9 scale (1 = highly susceptible, 9 = highly resistant)	R3
Pollen viability (Pollen shape 30, 60, 90 and 120 minutes)	Percentage of pollen grains with smooth, flat walls (0% = all rough pollen, 100% all flat pollen)	R1
Pollen viability (Pollen color 30, 60, 90 and 120 minutes)	Percentage of pollen grains with intense yellow color (0% = all pollen grains white, 100% = all pollen grains yellow)	R1
Herbicide injury	Percentage of plant tissue/leaf area injured over all plants in plot	7,14, 21, and 28 DAA^1^
Insect damage	1–9 scale (1 = highly susceptible; 9 = highly resistant)	V5 and R3
Stay green	1–9 scale (1 = all tissue green, 9 = no green tissue remaining)	R5
Plant height	cm	R5
Ear height	cm	R5
Root lodging	Percentage of plots in the plant leaning in the first 0.5 m above the soil surface	R5
Stalk lodging	Percentage of plants in the plot with stalks broken below the primary ear	R5
Days to Maturity	Number of days between planting and the day when 50% of the plants in the plot had reached physiological maturity	Harvest
Yield	Weight of grain harvested from plot (kg/ha)	Harvest

1 DAA – Days after application

The entries were composed of each unique combination of genotype and herbicide treatment. Entries included the GM corn hybrids MON 89034 × TC1507 × NK603 × DAS-40278–9 (PowerCore™ Enlist™), MON 89034 × TC1507 × NK603 (PowerCore™), and DAS-40278–9 (Enlist™), a non-GM near-isogenic hybrid, and the commercial non-GM reference hybrids 2B688 (Dow AgroSciences, São Paulo, SP, Brazil) and 30A68 (Morgan Seeds, São Paulo, SP, Brazil) (Table 2).

**TABLE 2.** Entries description.

Entries description
PowerCore™
Enlist™ sprayed sprayed with 2,4-D
PowerCore™ Enlist™ sprayed with 2,4-D+glyphosate;
Commercial non-GM reference hybrids 2B688
Commercial non-GM reference hybrids 30A68
Non-GM near-isogenic hybrid

MON 89034 × TC1507 × NK603 (PowerCore™) hybrid corn expresses 3 Bt proteins (Cry1A.105, Cry2Ab2, and Cry1F) that provide protection against stalk-boring and leaf-feeding lepidopteran pests (Siebert et al., 2012) and also provides tolerance to the herbicides glufosinate and glyphosate via expression of the CP4 EPSPS and PAT proteins e.

DAS-40278–9 (Enlist™) expresses the AAD-12 protein and provides tolerance to the herbicide 2,4-D. MON 89034 × TC1507 × NK603 × DAS-40278–9 (PowerCore™ Enlist™) hybrid corn expresses 3 Bt proteins (Cry1A.105, Cry2Ab2, and Cry1F) that provide protection against stalk-boring and leaf-feeding lepidopteran pests. In addition, this breeding stack provides tolerance to the herbicides glufosinate, glyphosate, 2,4-D, and certain aryloxyphenoxypropionate (AOPP) herbicides, which allows for the control of a broad spectrum of weeds with multiple herbicide modes of action. The combination of proven technologies in PowerCore™ Enlist™ hybrid corn maximizes a grower's herbicide choices and insect pest management options.

Plots were arranged in a randomized complete block design with 3 blocks at each field site. Plots were 8 rows wide by 8 m long with a row spacing of approximately 76 cm and were planted at a seeding rate of approximately 80 seeds per row (approximate seed spacing within each row was 10 cm). Crop production management practices to support optimum production, such as use of fertilizers, herbicides and insecticides, were applied to the entire plot area to produce a uniform crop, and irrigation was available when needed for optimum crop production.

Combinations of the herbicides 2,4-D, glyphosate, and haloxyfop were applied to entries that were tolerant to the herbicides (Table 2). The Enlist™ entry was sprayed with 2,4-D (1368 g ae/ha) before planting, at planting, and at the V2–V4 and V8–V10 growth stages and with haloxyfop at planting (182 g ae/L) and at the V2–V4 and V8–V10 growth stages (73 g ae/ha). The PowerCore™ Enlist™ entry was sprayed with 2,4-D + glyphosate (1170 + 1025 g ae/ha) before planting, at planting, and at the V2–V4 and V8–V10 growth stages, and with haloxyfop at planting (182 g ae/L) and at the V2–V4 and V8–V10 growth stages (73 g ae/ha). Herbicides were applied in a carrier volume of 100 L/ha and the haloxyfop applications included mineral oil at 1% v/v.

As primary measure of comparison, paired contrasts (*t*-tests) were conducted between the non-GM near-isogenic hybrid and each GM entry. Following ANOVA, a false-discovery rate (FDR) procedure was used to adjust *P*-values for multiplicity (Benjamini and Hochberg, 1995); differences were considered to be statistically significant at *P* < 0.05. As a second method to compare and interpret results, across-location means of the GM entries were examined within the context of the ranges observed in the 2 commercial hybrids included in the study.

## RESULTS

The overall treatment effect, least squares mean, pooled standard error, range, *P*- value, and FDR-adjusted *P*-value are reported for each agronomic characteristic evaluated (Table 3). For the commercial reference hybrids, only the range is reported to put any statistically significant differences into context.

**TABLE 3.** Comparative analysis of the average for the agronomic attributes, across locations between the iso-hybrid and the genetically modified treatments PowerCore™ Enlist™ sprayed, PowerCore™ and Enlist™ sprayed.

Agronomic characteristic (units)	Treatment effect. (Pr > F)^a^	Non-GM near-isogenic hybrid Average ± SE Min – Max	PowerCore™ Average ± SE Min – Max (P value; P Adjust.) ^b^	Enlist™ Average ± SE Min – Max (P value; P Adjust.) ^b^	PowerCore™ Enlist™ Average ± SE Min – Max (P value; P Adjust.) ^b^	Reference range Min – Max^C^
initial stand (number of plants)	0.676	89 ± 31	79 ± 31	76 ± 31	71 ± 31	50–115
		48–131	45–120	46–108	11–151	
			(0.548; 0.887)	(0.447; 0.833)	(0.294; 0.794)	
final stand (number of plants)	0.220	62 ± 19	62 ± 19	59 ± 19	65 ± 19	44–84
		37–84	40–81	36–81	43–84	
			(0.824; 1.0)	(0.205; 0.794)	(0.313; 0.794)	
time to silking (days after planting)	0.500	57 ± 3	57 ± 3	57 ± 3	57 ± 3	56–61
		54–61	54–60	54–59	54–60	
			(0.651; 0.89)	(0.391; 0.794)	(0.651; 0.887)	
pollen shape: 60 minutes (%)	0.835	86 ± 12	87 ± 12	83 ± 12	83 ± 12	50–100
		70–100	80–95	70–100	70–100	
			(0.886; 1.0)	(0.621; 0.887)	(0.621; 0.887)	
pollen shape: 90 minutes (%)	0.476	81 ± 16	78 ± 16	72 ± 16	70 ± 16	40–100
		70–95	60–90	50–90	50–90	
			(0.661; 0.887)	(0.274; 0.794)	(0.213; 0.794)	
pollen shape: 120 minutes (%)	0.603	68 ± 21	68 ± 21	58 ± 21	58 ± 21	40–95
		50–85	40–85	30–85	30–85	
			(0.937; 1.0)	(0.343; 0.794)	(0.376; 0.794)	
pollen color: 30 minutes (%)	0.584	50 ± 49	52 ± 49	50 ± 49	51 ± 49	0–100
			0–100	0–100	0–100	
		0–100	(0.287; 0.794)	(1.0; 1.0)	(0.564; 0.887)	
pollen color: 60 minutes (%)	0.500	50 ± 48	54 ± 48	51 ± 48	53 ± 48	5–100
		0–100	5–100	0–100	0–100	
			(0.209; 0.794)	(0.771; 0.985)	(0.409; 0.796)	
pollen color: 90 minutes (%)	0.500	54 ± 45	56 ± 45	54 ± 45	56 ± 45	5–100
		5–100	10–100	5–100	10–100	
			(0.308; 0.794)	(1.0; 1.00)	(0.308; 0.794)	
pollen color: 120 minutes (%)	0.500	56 ± 43	59 ± 43	56 ± 43	58 ± 43	5–100
		10–100	15–100	10–100	15–100	
			(0.264; 0.794)	(1.0; 1.0)	(0.379; 0.794)	
plant vigor (1–3 scale: 1 = poor vigor, 3 = excellent vigor)	0.500	2 ± 0	2 ± 0	2 ± 0	2 ± 0	2–3
		2–3	2–3	2–2	2–3	
			(0.514; 0.865)	(0.514; 0.865)	(0.514; 0.865)	
insect damage: V5 (1–9 scale: 1 = susceptible, 9 = resistant)	0.120	5 ± 2	8 ± 2	7 ± 2	8 ± 2	4–8
		3–7	6–9	4–9	6–9	
			(0.049; 0.794)	(0.187; 0.794)	(0.049; 0.794)	
insect damage: R3 (1–9 scale, 1 = susceptible, 9 = resistant)	0.096	6 ± 1	8 ± 1	7 ± 1	8 ± 1	4–8
		4–7	3–9	4–9	6–9	
			(0.076; 0.794)	(0.095; 0.794)	(0.028; 0.794)	
plant height (cm)	0.500	226 ± 25	228 ± 25	224 ± 25	223 ± 25	200–265
		200–257	200–260	200–252	200–252	
			(0.686. 0.89)	(0.583. 0.887)	(0.326. 0.794)	
ear height (cm)	0.310	110 ± 2	107 ± 2	113 ± 2	107 ± 2	106–119
		105.0–114	95–111.0	111.0–115.5	101.0–114	
			(0.350; 0.794)	(0.415; 0.796)	(0.381; 0.794)	
stalk lodging (%)	0.500	11 ± 8	5 ± 8	11 ± 8	0 ± 8	0–1
		0–50	0–25	0–30	0–0	
			(0.498; 0.865)	(1.0; 1.0)	(0.238; 0.794)	
root lodging (%)	0.583	8 ± 5	3 ± 5	5 ± 5	3 ± 5	0–5
		0–15	0–5	0–10	0–5	
			(0.318; 0794)	(0.566; 0.887)	(0.262; 0.794)	
yield (kg/ha)	0.288	4718 ± 435	5353 ± 435	4968 ± 435	5927 ± 435	7519–10713
		3338–6046	3933–6051	3470–6169	4616–7112	
			(0.311; 0.794)	(0.664; 0.887)	(0.104; 0.794)	

**Notes and Abbreviations**: Enlist™ entry was sprayed with 2,4-D; PowerCore™ Enlist™ entry was sprayed with 2,4-D+glyphosate;

a Treatment effect estimated using F test.

b Comparison of PowerCore™, Enlist™ and PowerCore™ Enlist™ with the isoline using t-tests; Adj. P = P-values adjusted using False Discovery Rate (FDR) procedure; P-values <0.05 were considered significant and are denoted with *.

C Min–Max represents range of individual replicate plot values.

Analysis of variance (ANOVA) was not conducted on characteristics that did not contain sufficient variability in the results to analyze (pollen shape at 30 min, time to pollen shed, days to maturity, and disease incidence). Herbicide injury was not included in the statistical analysis because the non-GM hybrid was not sprayed with the herbicides 2,4-D, glyphosate, or haloxyfop. Across-location mean injury caused by 2,4-D, glyphosate, and haloxyfop in the PowerCore™ Enlist™ plots after the final (V8–V10) application was 4% at 7, 14, 21, and 28 d after application. No herbicide injury was observed after the previous applications in the PowerCore™ Enlist™ entry or after any of the applications in the PowerCore™ and Enlist™ entries.

## DISCUSSION

No significant overall treatment effect was observed between the non-GM near-isogenic hybrid and the GM treatments (sprayed and unsprayed) for of the agronomic characteristics assessed: initial stand, final stand, time to pollen silk, pollen color at 30, 60, 90, and 120 minutes, pollen shape at 60, 90, and 120 minutes, plant vigor, insect damage at V5 and R3, stay green, plant height, ear height, stalk lodging, root lodging and yield.

For insect damage at V5 and R3, the 2 GM entries containing the insect protection traits (PowerCore™) had significantly less insect damage compared with the non-GM near-isogenic hybrid based on pairwise un-adjusted *P*-values; however, the overall treatment effect and FDR-adjusted pairwise *P*-value was not significant. This numerically superior insect resistance may be due to the intended insect resistance traits in this entry and the inability of insecticide sprays to match this level of protection.

Mean results of all agronomic characteristics evaluated for the non-GM near-isogenic hybrid and GM corn treatments were within the range of the commercial corn reference hybrids, except root lodging for the non-GM near-isogenic hybrid, and stalk lodging for all entries except MON 89034 × TC1507 × NK603 × DAS-40278–9 which all fell above the reference range. Yield and stay green for all treatments fell below the reference range perhaps due to the level of introgression of elite characteristics into these GM lines.

Based on similarity of the GM hybrids to the non-GM near-isogenic hybrid and commercial reference lines, it is concluded that PowerCore™ Enlist™, PowerCore™ and Enlist™corn are agronomically equivalent to non-transgenic corn.

## CONCLUSION

The agronomic characteristics for PowerCore™ Enlist™, PowerCore™, and Enlist™ corn hybrids were statistically indistinguishable from the non-GM near-isogenic hybrid. Most of the agronomic attributes were within the range of the commercial reference hybrids included in this study. These results support the agronomic equivalence of the GM entries and non-GM corn.
